# High sensitive troponin T and heart fatty acid binding protein: Novel biomarker in heart failure with normal ejection fraction?: A cross-sectional study

**DOI:** 10.1186/1471-2261-11-41

**Published:** 2011-07-05

**Authors:** Wilfried Dinh, Werner Nickl, Reiner Füth, Mark Lankisch, Georg Hess, Dietmar Zdunek, Thomas Scheffold, Michael Coll Barroso, Klaus Tiroch, Dan Ziegler, Melchior Seyfarth

**Affiliations:** 1 Department of Cardiology, Witten/Herdecke University, HELIOS Klinikum Wuppertal, Germany; 2 CoroVital, Institute for Sports Medicine, Germany; 3 Medical Department, University Mainz, Germany; 4 Roche Diagnostics GmbH, Mannheim, Germany; 5 Institute for Clinical Diabetology, German Diabetes Center at the Heinrich-Heine University, Leibniz Center for Diabetes Research, Düsseldorf, Germany; 6 Department of Metabolic Diseases, University Hospital, Düsseldorf, Germany

## Abstract

**Background:**

High sensitive troponin T (hsTnT) and heart fatty acid binding protein (hFABP) are both markers of myocardial injury and predict adverse outcome in patients with systolic heart failure (SHF). We tested whether hsTnT and hFABP plasma levels are elevated in patients with heart failure with normal ejection fraction (HFnEF).

**Methods:**

We analyzed hsTnT, hFABP and N-terminal brain natriuretic peptide in 130 patients comprising 49 HFnEF patients, 51 patients with asymptomatic left ventricular diastolic dysfunction (LVDD), and 30 controls with normal diastolic function. Patients were classified to have HFnEF when the diagnostic criteria as recommended by the European Society of Cardiology were met.

**Results:**

Levels of hs TnT and hFABP were significantly higher in patients with asymptomatic LVDD and HFnEF (both p < 0.001) compared to controls. The hsTnT levels were 5.6 [0.0-9.8] pg/ml in LVDD vs. 8.5 [3.9-17.5] pg/ml in HFnEF vs. <0.03 [< 0.03-6.4] pg/ml in controls; hFABP levels were 3029 [2533-3761] pg/ml in LVDD vs. 3669 [2918-4839] pg/ml in HFnEF vs. 2361 [1860-3081] pg/ml in controls. Furthermore, hsTnT and hFABP levels were higher in subjects with HFnEF compared to LVDD (p = 0.015 and p = 0.022).

**Conclusion:**

In HFnEF patients, hsTnT and hFABP are elevated independent of coronary artery disease, suggesting that ongoing myocardial damage plays a critical role in the pathophysiology. A combination of biomarkers and echocardiographic parameters might improve diagnostic accuracy and risk stratification of patients with HFnEF.

## Background

Nearly half of the patients with symptoms and signs of heart failure have a normal ejection fraction(EF) [1,2], a condition termed "heart failure with normal ejection fraction" (HFnEF). The overall mortality in patients with HFnNF is similar to that in patients with systolic heart failure (SHF) [1,3]. Furthermore, asymptomatic left ventricular diastolic dysfunction (LVDD), which is considered as a precursor of HFnEF, is a powerful and independent predictor of death [4]. Nevertheless, randomized trials in patients with HFnEF have failed to demonstrate a reduction in mortality [5]. This is presumably related to the considerable heterogeneity among patients with HFnEF and the lower proportion of specific heart failure related death in this population [6]. It is likely that the non-cardiovascular mortality in HFnEF patients contributes disproportionately to their all-cause mortality. Therefore, better characterization and accurate diagnosis of patients with HFnEF at greatest risk for heart failure related death would allow a more effective use of a specific therapy. In this regard, the diagnostic accuracy of echocardiography has been limited.

Circulating biomarkers have become increasingly important in diagnosing and risk stratifying patients with chronic heart failure (HF) [7,8].

N-terminal brain natriuretic peptide (NT-proBNP) has become an established diagnostic marker of heart failure and has been integrated in the guidelines [8,9], including diagnosis of HFnEF [10]. Recently, a highly sensitive commercial assay of cardiac troponin T (hsTnT) became available [11,12]. Using this assay, increased hsTnT levels were detected in the majority of patients with chronic systolic heart failure (SHF) [13] or ischemic heart disease, providing independent prognostic information with respect to heart failure admission and cardiovascular death [14,15].

Likewise, heart fatty acid binding protein (hFABP) has been reported to be associated with an increased risk of death in patients with SHF [16-18]. However, the use as a screening tool in subjects with LVDD or HFnEF remains to be established. This study sought to evaluate whether hsTnT and hFABP are elevated in patients with LVDD or HFnEF independent of coronary anatomy.

## Methods

### Study population

One hundred thirty consecutive hospitalized subjects referred to elective coronary angiography for the diagnostic workup of exercise intolerance, stable or suspected coronary heart disease (CAD) were enrolled in this study. Patients with the need for coronary revascularization either with angioplasty or coronary bypass surgery were excluded from further analysis. The protocol was approved by the local ethics committee, and signed informed consent was obtained from all patients. Inclusion criteria were scheduled coronary angiography, age 18-80 years and normal left ventricular ejection fraction (EF) ≥ 50%. Exclusion criteria were hypertrophic or infiltrative cardiomyopathy, moderate-to-severe valvular disease, atrial fibrillation or other severe arrhythmias, alcoholism, or serum-creatinine > 2.5 mg/dl. Considering the association between diabetes, HFnEF [19,20] and hsTnT release [21], we performed a standardized oral glucose tolerance test (oGTT, 75 g glucose) as previously described [22] in all patients without diabetes.

### Echocardiography

Echocardiography was performed using a standard ultrasound system (Vivid 7, General Electrics, Milwaukee, Wisconsin). Standard echocardiographic 2D measurements were performed according to current guidelines [23]. Conventional transmitral flow was measured with pw-Doppler. Early (E), late atrial (A) transmitral peak flow velocities and the ratio (E/A) were measured. Pulsed wave tissue Doppler imaging (TDI) was performed at the junction of the left ventricular (LV) wall with the septal and lateral mitral annulus and three consecutive beats were averaged. Early diastolic velocities (E″medial, E″ lateral) were recorded; the mean value (E″ average) from E″ at the medial and lateral mitral annulus was determined. Ratios of E/E″ medial, E/E″ lateral and average E/E″ ratio were calculated. Patients were classified to have HFnEF when the diagnostic criteria as recommended by the European Society of Cardiology were met [10]. In summary, there criteria include an E/E″ratio > 15 and NT-proBNP levels > 220 pg/ml. Mild asymptomatic left ventricular diastolic dysfunction (LVDD) was defined as E″ medial < 8 cm/s, the E/E″ medial ratio 8-15, NT-proBNP levels < 220 pg/ml and an E/A ratio < 0.8 cm/s.

### Biomarker

Before coronary angiography, blood samples were collected. The plasma supernatant was separated and stored frozen at -80°C until analysis. All laboratory measurements on the new hsTnT, NT-proBNP and hFABP were performed in the research laboratory of Roche Diagnostics, Penzberg, Oberbayern.

Troponin T concentrations were measured with high sensitive troponin T reagents on an Elecsys 2010 analyzer (Roche Diagnostics, Indianapolis, Indiana), with an analytical measurement range of 3-10000 ng/L or pg/mL. In studies performed with the Elecsys Troponin T high sensitive assay involving 533 healthy volunteers, the upper reference limit (99th percentile) for troponin T was 14 ng/L (pg/mL), 95% confidence interval 12.7-24.9 ng/L (pg/mL).

Heart acid fatty binding protein levels were measured on a human H-FABP ELISA kit (Hycult biotech) with an analytical measurement range of 102 to 25.000 pg/ml.

Details of NT-proBNP measurements have been described previously [24].

### Statistical Analysis

All analyses were performed using SPSS statistical software (SPSS 19.0, Chicago, IL). The data are presented as median with 25^th^/75^th ^percentiles (interquartile range) for continuous variables or absolute number (%) for categorical variables unless otherwise specified. Log transformed values were used for analysis as appropriate. A p value < 0.05 was considered statistically significant. The Mann-Whitney U-test was used to analyze differences between the medians of two groups and the Kruskal Wallis test to test the equality of medians among more than two distinct groups. Fisher″s Test was used for the comparison of two sets of binary variables and the χ2 test to evaluate differences in proportions in more than 2 sets of categorical variables.

High sensitive troponin T and hFABP were compared across subjects with normal diastolic function, mild LVDD and HFnEF by the Jonckheere-Terpstra test. We used the Spearman rank correlation to identify variables associated with biomarkers. Multiple linear regression analysis was applied to identify factors that were independently associated with hsTnT and hFABP levels.

## Results

### Patient characteristics

We included 130 patients with normal EF ≥ 50% (median age 67 [59-73] years, 49% woman) in the study, 62% of whom had stable CAD (defined as coronary stenosis > 50% in ≥ 1 coronary artery) without the need for coronary revascularization. The study group was subdivided as having either HFnEF (n = 49), asymptomatic left ventricular diastolic dysfunction (LVDD, n = 51) and normal diastolic function (controls, n = 30). An oGTT was performed in 95 individuals, of whom 38 (29%) had a normal glucose tolerance (NGT), 34 (26%) had impaired glucose tolerance and 23 (18%) had a new detected diabetes mellitus. Thirty-five patients had a history of type 2 diabetes mellitus before inclusion; therefore, 58 (45%) individuals included in the study were identified with type 2 diabetes mellitus (T2DM).

The clinical characteristics in patients classified as to the presence or absence of LVDD or HFnEF are shown in table 1, the laboratory data and parameter of cardiac assessment are highlighted in table 2.

**Table 1:** Clinical characteristics

	Normal DF (n = 30)	mild LVDD (n = 51)	HFnEF (n = 49)	p-value	All (n = 130)
**Clinical variables**					
Age (years)	60 (50-66)	65 (57-69)	72 (67-76)	<0.001*	67 (59-73)
Female gender	15 (50)	21 (41)	28 (57)	0.278	64 (49)
BMI (kg/m^2^)	26 (24-32)	27 (25-31)	28 (25-31)	0.236	27 (25-32)
Systolic BP (mmHg)	123 (110-130)	130 (126-142)	138 (130-140)	<0.001*	130(122-140)
Diastolic BP (mmHg)	76 (70-80)	80 (76-86)	80 (70-84)	0.023*	80(70-83)
CAD	16 (53)	31 (61)	34 (69)	0.346	81 (62)
Previous MI	7 (23)	7 (14)	14 (29)	0.189	28 (22)
Previous stroke	0	2 (4)	2 (4)	0.339	4 [39]
Previous PTCA	14 (46)	25 (496)	25 (51)	0.905	64 (49)
**Cardiovascular risk factors**					
Treated hypertension	23 (77)	46 (90)	47 (96)	0.010*	116 (89)
Smoking	7 (23)	8 (16)	6(12)	0.041*	21 (16)
Family history CAD	15 (50)	29 (57)	20 (42)	0.669*	64 (50)
Hyperlipidaemia	14 (46)	38 (74)	33 (67)	0.037*	85 (65)
**Glucose metabolism status**					
NGT	13 (43)	13 (25)	12 (24)	0.013*	38 (29)
IGT	11 (37)	13 (25)	10 (20)		34 (26)
New detected T2DM	3 (10)	8 (16)	12 (24)		23(18)
Known T2DM	3 (10)	17 (33)	15 (31)		35 (29)
**Medications**					
ACE inhibitor	17 (56)	29 (57)	34 (69)	0.359	80 (61)
AT1 blocker	2 (7)	8 (16)	11 (22)	0.179	21 (16)
Diuretics	5 (17%)	12 (24)	24 (49)	0.003*	41 (32)
Ca^2+ ^blocker	4 (13)	6 (12)	17 (34)	0.011*	27 (21)
ß-Blocker	21 (70%)	32 (63)	42 (86)	0.032*	95 (73)
Insulin therapy	0	8 (20)	6 (15)	0.128	14 (11)
OAD	1 (6)	13 (34)	5 (12)	0.012*	19 (15)

Values are median (interquartile range) or n (%), * statistically significant (p < 0.05). BMI = Body mass index, BP = blood pressure, CAD = Coronary Artery Disease, CABG = coronary bypass graft, DF = diastolic function, HFnEF = heart failure with normal ejection fraction, IGT = impaired glucose tolerance, LVDD = left ventricular diastolic dysfunction, NGT = normal glucose tolerance, OAD = oral anti-diabetic therapy, PTCA = percutaneous coronary angioplasty, T2DM = type 2 diabetes mellitus

**Table 2:** Laboratory data and echocardiographic parameter of cardiac assessment

	Normal DF (n = 30)	mild LVDD (n = 51)	HFnEF (n = 49)	p-value
**Biomarker**				
NT-proBNP (pg/ml)	89 (43-120)	81 (54-118)	444 (251-937)	<0.001*
hsTnT (pg/ml)	< 3 (< 3-6.4)	5.6 (< 3-9.8)	8.5 (3.9-17.5)	0.001*
hFABP (pg/ml)	2361 (1860-3081)	3029 (2533-3761)	3669 (2918-4839)	<0.001*
**Routine parameter**				
LDL (mg/dl)	106 (92-130)	106 (84-134)	111 (84-137)	0.927
HDL (mg/dl)	53 (45-68)	53 (39-63)	49 (42-60)	0.823
Triglyceride (mg/dl)	125 (100-210)	146 (103-233)	152 (115-206)	0.762
Creatinin (mg/dl)	0,9 (0,7-1,0)	0,9 (0,8-1,0)	0,9 (0,8-1,2)	0.050
Hba1c (%)	5,7 (5,5-6,1)	6,1 (5,8-7,0)	6,2 (5,7-6,6)	0.004*
**Systolic function**				
Ejection fraction (%)	63(60-67)	67 (61-71)	67 (63-73)	0.103
GLS ( -,%)	19,0 (19,9-17,3)	20,3 (21,8-16,9)	18,6 (21,2-16,5)	0.323
**LV geometry**				
LVEDD (mm)	43 (41-48)	43 (39-47)	45 (40-48)	0.413
LVMi (g/m^2^)	75 (64-97)	84 (68-104)	100 (76-135)	0.011*
**Diastolic function**				
LA- Index (ml/m^2^)	27,5 (23,9-29,2)	28,1 (23,7-31,1)	39,1 (34,2-49,1)	<0.001*
E (cm/s)	60 (50-60)	60 (60-70)	70 (60-90)	<0.001*
A (cm/s)	70 (50-70)	80 (70-90)	80 (70-90)	<0.001*
E/A ratio	0,86 (0,71-1,18)	0,78 (0,71-0,89)	0,88 (0,77-1,25)	0.055
E' septal (cm/s)	8,0 (7,1-8,9)	5,9 (5,1-7,1)	5,6 (4,8-6,2)	<0.001*
E' lateral (cm/s)	10,4 (8,8-11,6)	8,2 (6,7-9,2)	7,3 (5,8-9,0)	<0.001*
E/E' septal ratio	7,1(6,2-7,7)	10,6 (8,8-12,2)	12,8 (10,9-16,4)	0.001*
E/E' average ratio	6,50(5,6-6,9)	9,0 (7,8-10,5)	11,2 (9,5-14,5)	<0.001*

Values are median (interquartile range). * Statistically significant (p < 0.05). A = late diastolic transmitral inflow velocity, EF = ejection fraction. DF = diastolic function, GLS = global longitudinal strain, LA = left atrial, E = early diastolic transmitral inflow velocity, E″ septal = early diastolic tissue doppler velocity septal, E″lateral = early diastolic tissue doppler velocity lateral, HFnEF = heart failure with normal ejection fraction, hFABP = heart fatty acid binding protein, hsTnT = high sensitive troponin T, LVEDD = left ventricular end-diastolic diameter, LV = left ventricular, LVMi = left ventricular muscle mass index, NT-proBNP = amino-terminal pro-B-type natriuretic peptide.

### High sensitive troponin T, hFABP and diastolic function

**Figure 1:**
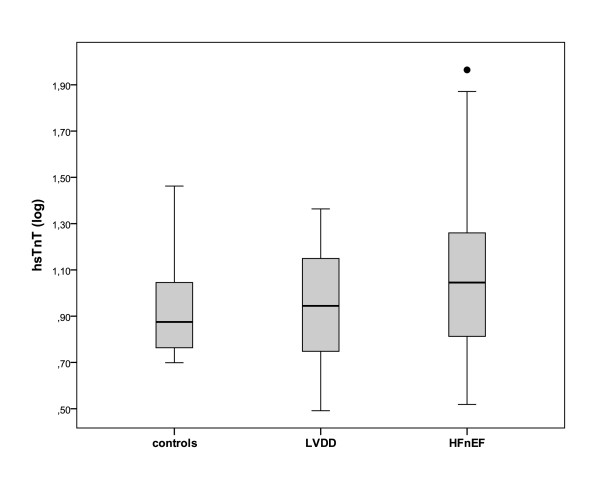
**High sensitive troponin T (hsTnT) levels plotted against diastolic function in patients with normal diastolic function (controls), mild asymptomatic left ventricular diastolic dysfunction (LVDD) and heart failure with normal ejection fraction (HFnEF)**. hsTnT levels are log transformed and presented as box (25th percentile, median, 75th percentile) and whiskers plots. Upper outliers are presented as black dots (>1.5 times box high).

**Figure 2:**
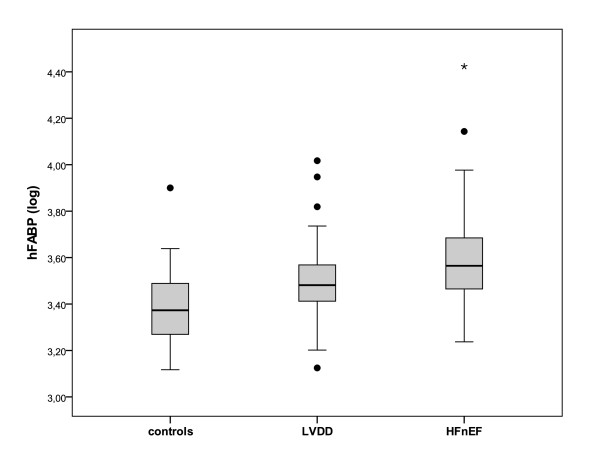
**Heart fatty acid binding protein (hFABP) levels plotted against diastolic function in patients with normal diastolic function (controls), mild asymptomatic left ventricular diastolic dysfunction (LVDD) and heart failure with normal ejection fraction (HFnEF)**. hFABP levels are log transformed and presented as box (25th percentile, median, 75th percentile) and whiskers plots. Upper and lower outliers are presented as black dots (>1.5 times box high); asterisks indicates extreme cases (>3 times of box high).

High sensitive troponin T and hFABP levels increase significantly from controls to asymptomatic LVDD and HFnEF (both p < 0.001, figure 1 and 2).

Furthermore, hsTnT and hFABP levels were higher in subjects with HFnEF compared to asymptomatic LVDD (p = 0.015 and p = 0.022, respectively). In multivariate analysis including age, sex, CAD, EF, body mass index and diabetes, the presence of HFnEF remains the only factor significantly associated with hsTnT levels (p = 0.009). Furthermore, hFABP was significantly higher in the asymptomatic LVDD group compared to those with normal diastolic function (3029 [2533-3761] pg/ml in LVDD vs. 2361 [1860-3081] in controls; p = 0.007), whereas hsTnT was not significantly different between the LVDD and controls (p = 0.068).

Excluding subjects with CAD, we found that hsTnT was detectable, at a level of 3.0 pg/ml or greater, in 87% of HFnEF patients, in 65% of the LVDD group, and in 36% of the control group subjects (p = 0.017). Furthermore, hsTnT was detectable above the upper reference limit of 14.0 pg/ml in 33% of HFnEF patients, in 15% of the LVDD group, and in 0% of the control group subjects (p = 0.05). Overall, in subjects without CAD, hsTnT and hFABP levels remain significantly associated with the presence and severity of diastolic dysfunction (p < 0.001, table 3).

**Table 3:** Laboratory data in subjects with or without stable coronary artery disease

	Normal DF	mild LVDD	HFnEF	p-value
**No CAD**	**n = 14**	**n = 20**	**n = 15**	
NT-proBNP (pg/ml)	66 (38-91)	90 (56-116)	381 (236-1147)	<0.001*
hsTnT (pg/ml)	< 3 (< 3-5.6)	4.5 (< 3-8.9)	8.5 (5.4-18.7)	0.001*
hFABP (pg/ml)	2066 (1822-2432)	3138 (2637-3818)	3710 (3126-8354)	<0.001*
**CAD**	**n = 16**	**n = 31**	**n = 34**	
NT-proBNP (pg/ml)	95 (55-137)	78 (54-122)	481 (253-4685)	<0.001*
hsTnT (pg/ml)	< 2.85 (< 3-8.7)	6.9 (< 3-10.0)	9.2 (3.6-17.5)	0.023*
hFABP (pg/ml)	3017 (2210-3661)	2956 (2533-3340)	3390 (2825-4685)	0.048*

Values are median (interquartile range. CAD = coronary artery disease, hFABP = heart fatty acid binding protein; HFnEF = heart failure with normal ejection fraction; hsTnT = high sensitive troponin T, LVEDD = left ventricular end-diastolic diameter; NT-proBNP = amino-terminal pro-B-type natriuretic peptide. * Statistically significant (p < 0.05).

Overall, in subjects without CAD, hsTnT and hFABP levels remain significantly associated with the presence and severity of diastolic dysfunction (p < 0.001, table 3).

The relationship between hsTnT and hFABP quartiles, cardiac assessment and NT-pro-BNP levels is shown in table 4 and 5. Particularly among the association with echocardiographic parameters of diastolic dysfunction, hsTnT and hFABP levels were significantly increasing across the E/E″ ratio, a parameter indicative for elevated ventricular filling pressures. There was a weak linear correlation between NT-proBNP levels with hsTnT (r = 0.331, p < 0.001) and hFABP levels (r = 0.330, p < 0.001).

**Table 4:** Parameter of cardiac assessment according to hsTnT quartiles

	1rd Quartile	2nd Quartile	3rd Quartile	4th Quartile	p- value
**hsTnT (pg/ml) Systolic function**	<3 (n = 44)	<3-5.6 (n = 18)	5.7-11.3(n = 37)	11.3-92.1 (n = 31)	
Ejection fraction (%)	66 (62-72)	62 (57-69)	66 (63-70)	65 (61-74)	0.267
GLS ( -,%)	19,8 (21,6-18,0)	19,2 (21,0-16,8)	18,9 (21,4-16,6)	18,3 (21,1-16,0)	0.477
**LV Geometry**					
LVEDD (mm)	44 (41-48)	43 (42-50)	45 (39-48)	43 (40-47)	0.804
LVMi (g/m^2^)	81 (68-89)	94 (74-121)	90 (70-107)	107 (69-138)	0.013*
**Diastolic function**					
LA- Index (ml/m^2^)	28 (23-34)	30 (24-33)	30 (27-37)	35 (29-39)	0.012*
E/A ratio	0,86 (0,75-1,20)	0,75 (0,68-0,85)	0,80 (0,71-1,00)	0,87 (0,75-1,00)	0.128
E' septal (cm/s)	7,3 (5,3-8,0)	6,2 (4,7-7,3)	5,9 (5,3-7,1)	5,7 (4,5-6,2)	0.004*
E' lateral (cm/s)	9,0 (7,2-10,43)	9,2 (6,1-10,8)	8,1 (6,7-9,1)	7,2 (5,6-8,2)	0.025*
E/E' septal ratio	9,0 (7,5-12,1)	10,4 (9,18-11,6)	11,2 (8,3-13,1)	11,8 (8,9-15,7)	0.023*
E/E' average ratio	7,83 (6,60-9,76)	8,89 (7,16-10,59)	9,45 (7,72-12,05)	10,4 (8,15-13,37)	0.015*
**Laboratory**					
NT-proBNP (pg/ml)	104 (49-166)	102 (52-223)	150 (93-265)	261 (78-926)	0.005*

Values are median (interquartile range). * Statistically significant (p < 0.05). A = late diastolic transmitral inflow velocity, EF = ejection fraction. GLS = global longitudinal strain, LA = left atrial, E = early diastolic transmitral inflow velocity, E″septal = early diastolic tissue doppler velocity septal, E″lateral = early diastolic tissue doppler velocity lateral, hsTnT = high sensitive troponin T, LV = left ventricular, LVEDD = left ventricular enddiastolic diameter, LVMi = left ventricular muscle mass index, NT-proBNP = amino-terminal pro-B-type natriuretic peptide.

**Table 5:** Parameter of cardiac assessment according to hFABP quartiles

	1rd Quartile	2nd Quartile	3rd Quartile	4th Quartile	p-value
**hFABP (pg/ml) Systolic function**	0-2405 (n = 33)	2406-3057 (n = 34)	3057-3908 (n = 32)	>3908 (n = 32)	
EF (%)	64 (60-68)	67 (61-72)	64 (62-70)	68 (63-74)	0.103
GLS ( -,%)	19.0 (20.6-17.0)	18.5 (21.4-16. 2)	19.0 (21.2-16.6)	20.3 (22.4-17. 3)	0.323
**LV Geometry**					
LVEDD (mm)	43 (40-48)	45 (40-49)	44 (41-51)	43 (40-47)	0.413
LVMi (g/m^2^)	76 (66-103)	94 (74-107)	83 (63-104)	97 (71-118)	0.011*
**Diastolic function**					
LA- Index (ml/m^2^)	27 (23-30)	28 (24-35)	31 (26-37)	36 (29-40)	<0.001*
E/A ratio	0.86 (0.75-1.00)	0.82 (0.75-1.00)	0.86 (0.71-1.11)	0.88 (0.71-1.13)	0.055
E' septal (cm/s)	7.2 (5.5-8.0)	5.8 (5.3-6.6)	6.2 (5.2-7.3)	5.6 (4.4-6.5)	<0.001*
E' lateral (cm/s)	10.3 (6.8-11.0)	8.1 (7.0-9.0)	8.9 (7.6-9.6)	6.8 (5.5-8.7)	<0.001*
E/E' septal ratio	8.3 (7.5-10.6)	10.8 (8.6-12.3)	11.2 (8.5-13.2)	12.3 (10.3-15.7)	<0.001*
E/E' average ratio	6.84 (6.47-8.77)	9.06 (7.80-10.70)	9.46 (7.59-10.71)	10.94 (9.33-14.51)	<0.001*
**Laboratory**					
NT-proBNP (pg/ml)	71 (39-197)	119 (62-201)	152 (95-257)	294 (98-572)	<0.001*

Values are median (interquartile range). * Statistically significant (p < 0.05). A = late diastolic transmitral inflow velocity, EF = ejection fraction. GLS = global longitudinal strain, LA = left atrial, E = early diastolic transmitral inflow velocity, E″septal = early diastolic tissue doppler velocity septal, E″lateral = early diastolic tissue doppler velocity lateral, hFABP = heart fatty acid binding protein, LV = left ventricular, LVEDD = left ventricular enddiastolic diameter, LVMi = left ventricular muscle mass index, NT-proBNP = amino-terminal pro-B-type natriuretic peptide.

In contrast to diastolic function parameter, left ventricular ejection fraction and the global longitudinal strain values, a very sensitive tool to detect systolic dysfunction disregarding a normal EF, were not associated with hsTnT or hFABP levels (both p > 0.05).

## Discussion

We have demonstrated for the first time that hsTnT and hFABP plasma levels are associated with the diagnosis of HFnEF. The association is in proportion to the severity of the disease. Furthermore, hFABP was significantly different in subjects with normal DF and asymptomatic LVDD, whereas whereas hsTnT was not significantly different between the LVDD and controls. Both hsTnT and hFABP levels correlate significantly with multiple echocardiographic criteria implemented in guidelines for the diagnosis and classification of LVDD and HFnEF.

### High sensitive troponin T

The recent introduction of a new generation hsTnT has not only improved the early diagnosis of acute coronary syndromes, but also suggested that there are several causes for troponin T release other than myocardial ischemia. Particularly patients with SHF were found to have detectable levels of hsTnT with a persistent relationship between magnitude and outcome. In several cohorts of patients with SHF, the magnitude of troponin elevation has been correlated with the severity of the disease and with adverse outcomes [25,26]. The Val-HeFT trial [13] showed an almost linear increase in the risk of adverse clinical event with hsTnT concentration in patients with SHF, even in a range of very low concentrations that could not be measured with the traditional assay. In this trial, measurement of hsTnT adds to the prognostic information provided by natriuretic peptides alone. Patients with both cardiac markers elevated had a worse prognosis than those with a single elevated marker.

Furthermore, in the general population, hsTnT was associated with structural heart disease and subsequent risk for all-cause mortality [27]. A recent study has shown that low levels of hsTnT are associated with new-onset heart failure and cardiovascular death in older adults >65 years without underlying cardiovascular disease, independent of other risk factors [28], and a large observation study in Europe has shown an association between low levels of circulating troponin T and the future development of HF in completely asymptomatic subjects [29]. We were able to demonstrate a strong association between hsTnT and the diagnosis of HFnEF, independent of CAD. Therefore, analogous to SHF, we hypothesize that hsTnT might improve diagnostic accuracy and risk stratification in HFnEF.

### Heart fatty acid binding protein

Heart fatty acid binding protein is abundant in the cytosol of cardiomyocytes and is released when cell surface membrane is injured [30]. In advanced SHF, hFABP levels are increased because of the leakage of cytosolic proteins from cardiomycates affected by the ongoing myocardial damage [17,24,31,32]. Circulating levels of hFABP have a prognostic value regarding the future deterioration of congestive heart failure in patients with dilated cardiomyopathy [16,33], and persistently increased serum concentrations of hFABP predict adverse clinical outcomes in patients with SHF [16]. Our data show a significant association between hFABP and the severity of diastolic dysfunction.

In contrast to hsTnT, hFABP was significantly increased in the asymptomatic LVDD group compared to controls. hFABP is a cytosolic protein, whereas troponin is a myofibrillar protein with a cytosolic pool estimated at only 6% to 8% [34]. A reversible myocyte injury resulting in increased membrane permeability would cause an early hFABP release, while a more extensive injury must occur before significant amounts of troponin are released. LVDD, which is considered as a precursor of HFnEF, carries a substantial risk for the subsequent development of HFnEF and reduced survival, even when it is asymptomatic [4]. Considering the large number of patients at risk for or with asymptomatic LVDD, early identification of LVDD may provide an opportunity to manage the underlying cause and prevent progression to symptomatic diastolic heart failure. Accordingly, hsFABP may be a more sensitive and reliable indicator of low-level myocardial damage in LVDD, especially when used together with troponins [16,17].

### Pathophysiological considerations

Elevated hsTnT and hFABP levels in patients with HFnEF may suggest ongoing myocardial damage at a very low rate [31], indicating that these biomarkers may serve as a marker for the progression of heart failure [35]. In our study, hsTnT and hFABP were increased in patients with HFnEF independent of CAD. Hence, this phenomenon seems to be independent of an ischemic injury. Stretching of myocytes might lead to leakage of troponins and hFABP by transient loss of cell membrane integrity without cell death [36]. This reversible damage may contribute to the increase in circulating cardiac troponins caused by irreversible damage of myocytes [26]. Nevertheless, persistently elevated hsTnT and hFABP values in HFnEF patients should lead to an evaluation for ischemic heart disease, if not already performed.

According to the diagnostic criteria as recommended by the European Society of Cardiology in 2007 [10], N-terminal brain natriuretic peptide (NT-proBNP) is regarded as the preferred biomarker for the detection of HFnEF. Nevertheless, in our study, the correlation between NTproBNP and hsTnT or hFABP was only moderate, suggesting that BNP and specific myocardial proteins convey different and complementary features of the pathophysiologic process. The former is released in response to the pressure overload and the latter reflects structural alterations in the myocardium and ongoing myocardial damage. In patients with SHF, it has been reported that the combined measurement of BNP and troponin can predict adverse cardiac events [37]. Consequently, these biomarkers may provide different diagnostic or prognostic information in patients with HFnEF.

### Clinical considerations

In daily clinical practice, although specific recommendations have been proposed [10,38], affirmation of HFnEF is challenging because the HFnEF population is heterogeneous, and HFnEF is probably not necessarily a single entity. This implies a high risk for either a false positive or false negative diagnosis by the defining diagnostic criteria. Consequently, identification of potentially pathophysiologically distinct subgroups of HFnEF patients could advance diagnosis and therapy. Particularly, a test that identifies which patients with HFnEF are at increased risk for cardiovascular events would be desirable. In this regard, changes in different biomarker levels in HFnEF are of scientific interest, as they reflect distinct disease mechanisms in heart failure.

## Limitations

Interpreting the present data is limited by the small number of the patients studied, resulting in a limited statistical power. Furthermore, the rates of CAD and cardiovascular risk factors were high in this study population. Therefore, the present results may not be readily representing the general population. Nevertheless, the association between diastolic function, hsTnT and hFABP remains significant after adjustment for CAD, glucose metabolism and hypertension as covariates into multivariate regression models. Furthermore, for risk stratification, follow-up and association of the biomarkers with clinical events is needed. Lastly, we did not perform serial measurements and only focused on baseline values. Accordingly, our cross sectional study design does not permit any conclusions on causality.

## Conclusions

This is the first study to show that circulating hsTnT and hFABP are elevated in patients with HFnEF independently of CAD. Nevertheless, the mechanisms of cardiac injury in HFnEF resulting in hsTnT and hFABP release need to be further elucidated.

Incorporation of a multimarker strategy, reflecting distinct pathophysiological mechanisms, may improve diagnostic accuracy and risk prediction in HFnEF beyond traditional risk indicators. Further studies assessing mortality and morbidity are needed to evaluate whether the use of hsTnT and hFABP can guide the identification of HFnEF at high risk.

## Competing interests

The authors declare that they have no competing interests.

## Authors' contributions

WD wrote manuscript, researched data, performed echocardiographic measurements and statistical analysis. WN researched data and contributed to discussion. RF performed echocardiographic measurements and ML reviewed manuscript and contributed to the discussion. GH and DZ performed laboratory analysis. MCB researched data, TS, KT, DZ and MS researched data, edited manuscript and contributed to discussion. All authors read and approved the final manuscript.

## Pre-publication history

The pre-publication history for this paper can be accessed here:

http://www.biomedcentral.com/1471-2261/11/41/prepub
